# Time course of collagen peak in bile duct-ligated rats

**DOI:** 10.1186/1471-230X-11-45

**Published:** 2011-04-28

**Authors:** Orhan Tarcin, Metin Basaranoglu, Veysel Tahan, Gülgün Tahan, Ilker Sücüllü, Nevin Yilmaz, Gagan Sood, Ned Snyder, Gilbert Hilman, Cigdem Celikel, Nurdan Tözün

**Affiliations:** 1 Acibadem Hospital, Istanbul, 34500, Turkey; 2 Division of Gastroenterology and Hepatology, Consulting, Ankara Yüksek Ihtisas Hospital Gastroenterology Clinic, Sihhiye, Ankara, 06420, Turkey; 3 Institute of Gastroenterology, Surgery Unit, Marmara University Istanbul, 34500, Turkey; 4 Division of Gastroenterology, Marmara University Istanbul, 34500, Turkey; 5 Gülhane Military Medical Faculty, Haydarpasa Educational Hospital Istanbul, 34500, Turkey; 6 University of Texas Medical Branch, Division of Pharmacology, Galveston/USA; 7 University of Texas Medical Branch, Division of Gastroenterology, Galveston/USA; 8 Division of Pathology Marmara University, Istanbul, 34500, Turkey; 9 Division of Gastroenterology Acıbadem University, Istanbul, 34500, Turkey

**Keywords:** Bile duct-ligated, rat, fibrosis, fibrogenesis, liver, collagen, reversible

## Abstract

**Background:**

One of the most useful experimental fibrogenesis models is the "bile duct-ligated rats". Our aim was to investigate the quantitative hepatic collagen content by two different methods during the different stages of hepatic fibrosis in bile duct-ligated rats on a weekly basis. We questioned whether the 1-wk or 4-wk bile duct-ligated model is suitable in animal fibrogenesis trials.

**Methods:**

Of the 53 male Wistar rats, 8 (Group 0) were used as a healthy control group. Bile duct ligation (BDL) had been performed in the rest. Bile duct-ligated rates were sacrificed 7 days later in group 1 (10 rats), 14 days later in group 2 (9 rats), 21 days later in group 3(9 rats) and 28 days later in group 4 (9 rats). Eight rats underwent sham-operation (Sham). Hepatic collagen measurements as well as serum levels of liver enzymes and function tests were all analysed.

**Results:**

The peak level of collagen was observed biochemically and histomorphometricly at the end of third week (P < 0.001 and P < 0.05). Suprisingly, collagen levels had decreased with the course of time such as at the end of fourth week (P < 0.01 and P < 0.05).

**Conclusion:**

We have shown that fibrosis in bile duct-ligated rats is transient, i.e. reverses spontaneously after 3 weeks. This contrasts any situation in patients where hepatic fibrosis is progressive and irreversible as countless studies performed by many investigators in the same animal model.

## Background

Hepatic fibrosis is characterized by massive deposition of extracelluler matrix components in the liver. There are differences on liver fibrogenesis due to etiological factors [1].Currently, there are no animal models that completely mimic the spectrum of the diseases seen in humans.

One of the most useful experimental fibrosis models is the "bile duct-ligated rats". There is substantial information on morphological changes in the liver of the rats after obstructive jaundice to date. In 1932, Cameron et al. described gross macroscopic features and light microscopical changes in bile duct-ligated rats by intervals from 1/2 hour to 9 weeks [2]. Trams et al. in 1957 and Cameron et al. in 1958 studied functional and structural disturbances in the bile duct-ligated rats' liver [3,4]. Then, several investigators studied serum enzymes derived from liver cell fraction in the bile duct-ligated rat in 1972 [5]. Later studies investigated the effect of biliary obstruction on bile flow and bile acid secretion [6]. Kountauras et al. surveyed prolonged bile duct obstruction as a new experimental model for cirrhosis in the rat and proposed that as a good model for hepatic fibrosis studies in 1984 [7].

However, none of the previous studies investigated hepatic collagen content during ongoing and progressive hepatic fibrosis after bile duct ligation (BDL). Our aim was to investigate the quantitative hepatic collagen content by two different methods in bile duct-ligated rats within the course of time.

## Methods

53 male Wistar rats, weighting 244.62 ± 5.03 (mean ± SE) were included in the study. Eight rats (Group 0) were used as a healthy control group without operation. Thirty-seven underwent BDL. These animals were sacrificed seven days later in group 1 (n = 10), 14 days later in group 2 (n = 9), 21 days later in group 3 (n = 9) and 28 days later in group 4 (n = 9).

Eight rats underwent sham-operation (Sham). This study was approved by the Marmara University, Animal Use and Care Committee. All the experimental procedure was performed in conformity with the Guiding Principles for Research Involving Animals. Sacrifice was performed by decapitation, and trunk blood was obtained for each. Serum samples were collected for aspartate aminotransferase (AST), alanine aminotransferase (ALT), alkaline phosphatase (ALP), total and direct bilirubin and gamma-glutamyl transpeptidase (GGT) measurements, and stored at -80°C.

### Bile duct-ligated rats

The operation was performed under ketamine anesthesia. A midline ventral incision was made through the linea alba. The duodenum was delivered through the incision in order to place the bile duct under the tension. Bile duct was isolated. Then, two ligatures were placed to the proximal portion and one ligature was placed to the distal part of the bile duct. The ligatures were tightened. Then, bile duct divided by the scissors in each. In Shamoperated group ligatures were withdrawn for leaving the bile duct intact. Then peritoneum, linea alba and skin were closed with silk.

### Liver tissue sampling

The left, middle and right lobes of each liver were explored. We obtained 0.5 × 0.5 × 1.5 cm thick slices from each lobe randomly. These slices were fixed in 10% buffered formalin and routinely processed and blocked into the parafin.

### Collagen content determination by biochemically

**Figure 1:**
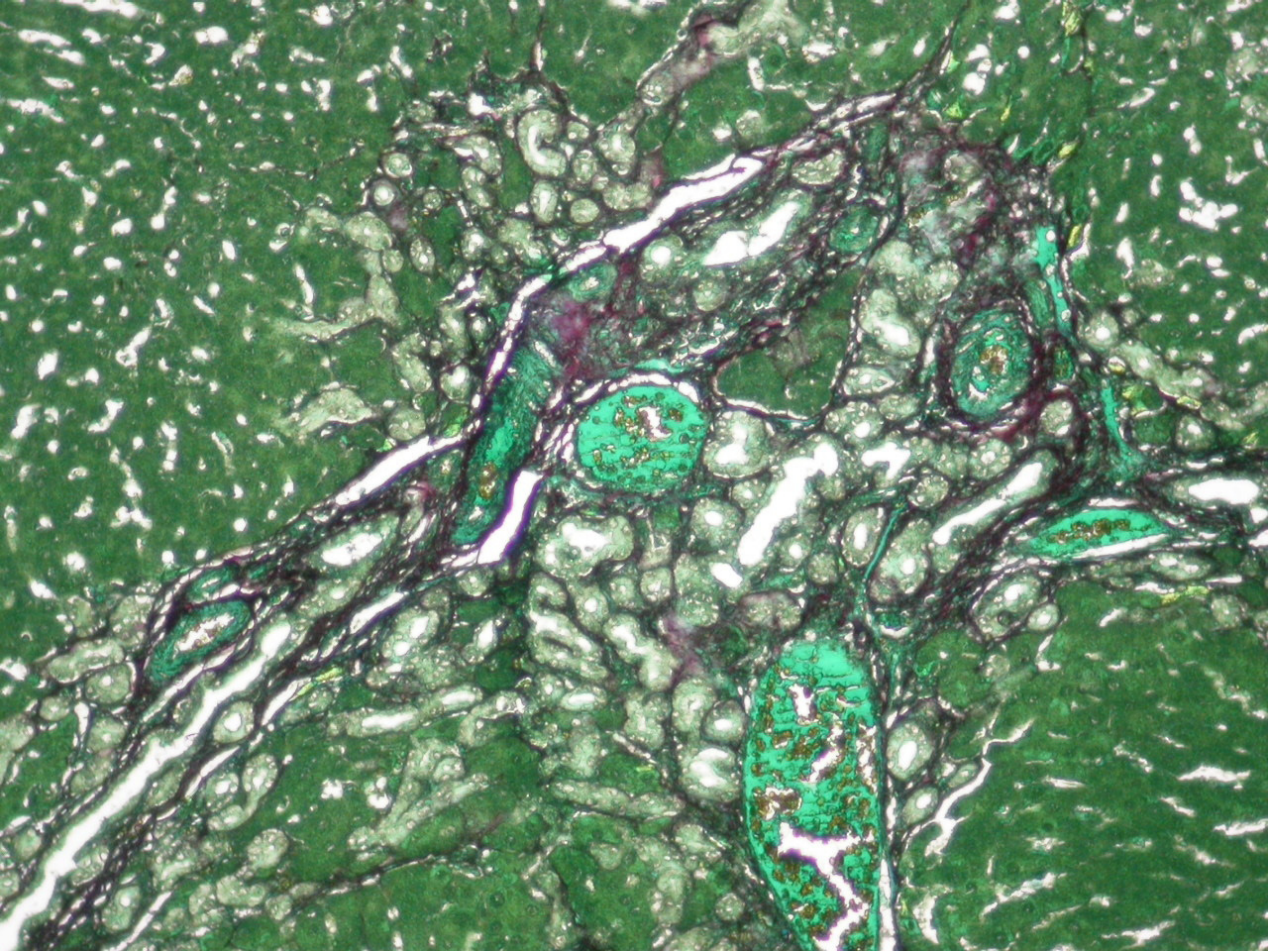
**Sirius red and fast green-stained liver sections showed prominent bile duct proliferation and fibrosis (× 20)**. Sirius red stained collagenous proteins. Fast green stained non-collagenous proteins.

The collagen content of the liver was assayed by the colorimetric method described by Lopez de Leon and Rojkind [8]. The principle is the coloring of collagenous protein by Sirius red (36554-8, 2610-10-8; Aldrich Chemical, Deisenhofen, Germany) and non-collagenous proteins by fast green (14280; MERCK, Darmstadt, Germany) (Figure 1). Fifteen micrometer-thick liver slices were taken from each paraffin block and layered on glass slides. Slices were deparaffinized after being incubated by xylol, xylol: ethanol(1:1), ethanol, water: ethanol, and water. The slides were stained with a saturated solution of picric acid in distilled water containing 0.01% fast green. Each section was kept out of the light and incubated at room temperature for 15 minutes. Then, sections were stained by a saturated solution of picric acid in distilled water containing 0.04% fast green and 0.1% of sirius red and incubated in the dark room temperature for 30 minutes. Then, samples were rinsed and transferred to a test tube containing 1 ml of 0.1% NaOH in absolute methanol (1:1). The tubes were gently mixed until the color was eluted completely. Absorbance of the eluted color was read at 540 and 605 nm by a Jasco V 50 UV-VIS spectrophotometer. Maximal absorbance of fast green is at 630 nm and 540 nm for Sirius red. Fast green exhibites a small absorbance (7.78%) at 540 nm. This interference remains constant at different concentrations of fast green. By contrast, sirius red has no absorbance at 630 nm. Therefore, sirius red does not interfere with non-collagenous protein determination. Collagen content of tissue was calculated using the formula below. It was described as in microgram collagen per milligram protein [8].

### Histomorphometric collagen measurement

Four μ slices were obtained from the paraffin blocks and stained by tricrome. We used a special image analysis software for histomorphometric measurements. It was referred to as "staining" that can perform automatic threshold which was written by an optical image analysis expert under a commercially available software 'Matlab^®^' (Natick, Massachusetts/USA). We used an automated histogram threshold method for measurements. We took the pictures by 40 × objective of an Olympus^® ^microscope (Hamburg/Germany) with a Nicon^® ^digital camera. 120 consecutive pictures from each slice were obtained. Each of these frames was represented by 1087 × 805 μm (0.875 mm^2^) area of the liver. 120 frames were used to reach 1 cm 2 of total surface. Image analysis software was run to automatically outline and to calculate the sum of green spectrum stained fibrotic area over red background. The collagenous areas were measured as a percentage of the total surface area of the liver.

### Histopathological investigations

**Figure 2:**
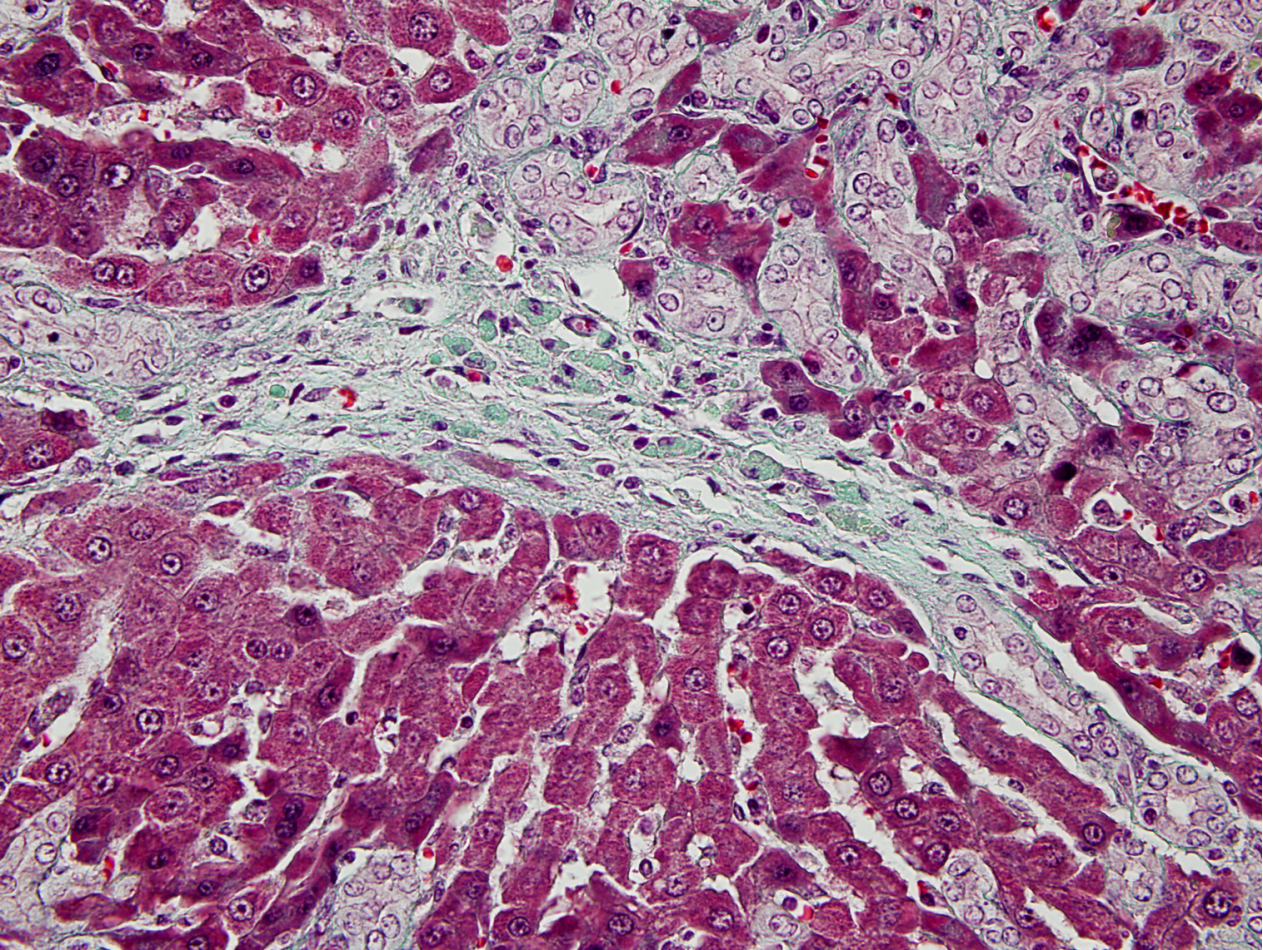
**Tricrome stained section showed portal-portal fibrosis (×40)**. Green areas were fibrotic and red areas were normal.

Five-micrometer liver sections were stained by hematoxylin-eosin and Masson's trichrom. We used Ishak fibrosis classification system for grading of necroinflammatory activity and staging of fibrosis [9]. We used a scoring system from 1 to 4 for grading of portal proliferation (Figure 2).

### Serum measurements

Serum AST and ALT levels were assessed by using commercial kits (Roche Diagnostics, GmbH, D-68298, Mannheim, Germany) in Roche-Hitachi Modular Autoanalyzer (Roche Diagnostics, GmbH, D-68298, Mannheim, Germany).Bayer Opera autoanalyzer was used to measure bilirubin levels by wavelength, 550 nm; temperature, 37°C; and instrument set for endpoint with sample blanking mode. Serum levels of GGT and ALP were measured by an Olympus AU 800 biochemical automated analyser.

### Statistic analysis

Kruskall-Wallis test was used to analyse the homogenity among the different groups of rats, Additionally, multiple post-hoc comparisons with Mann-Whitney U test was used. P value less than 0.05 was considered to be statistically significant. Calculation was performed by SPSS in v.11 program.

## Results

### Body weight

There were no significant differences about the body weights between the groups at any time. The animals slightly lost weight at the 1st week. They gained weight during the 2nd week. These changes never reached a difference between BDL rats, sham group and control group (Table 1)

**Table 1:** Mean body and liver weights were shown.

		Body weights at the beginning of the study		Body weights at the end of the study		Liver weight after sacrifice	
	**N**	**Mean**	**SD**	**Mean**	**SD**	**Mean**	**SD**

Control	8	254.71	*31.98*	293.28	35.71	9.78	*0.52*

Sham	8	239.42	*30.08*	298.00	38.71	10.44	*0.93*

Group 1	10	235.12	*20.35*	181.25*	35.48	10.76	*2.00*

Group 2	9	227.75	*34.52*	233.12	37.030	13.02**	*1.77*

Group 3	9	251.88	*29.78*	265.22	42.29	16.00**	*1.31*

Group 4	9	258.87	*38.09*	271.62	41.92	16.62**	3.77

*Body weight decreased significantly in group1 (P < 0.01).

**Liver weight increased in groups 2, 3 and 4 compared to control, sham and group 1 (P < 0.01).

### Liver weight

Liver weight increased at the 1st week, and continued to increase during the study. There was no significant difference between controls and group 1. By contrast, there were statistically significant differences between control groups and groups 2, 3 and 4 (P < 0.001) (Table 1).

### Histopathological findings

There were some differences in necroinflammatuary scores (NIS) and fibrosis scores (FS) among the groups. NIS and FS were higher in group 3 than in others (groups 1, 2 and 4). However, these differences were not significant, statistically (P > 0.05). Portal proliferation has been increased from the first week to the fourth week. The peak level of portal proliferation was observed at the end of the fourth week.

### Biochemical hepatic collagen measurement

**Figure 3:**
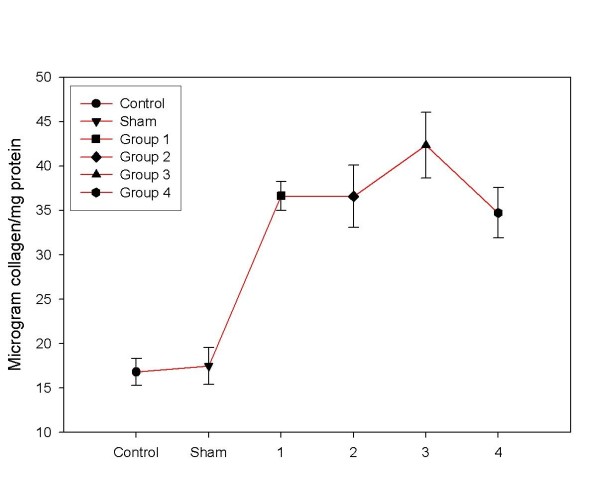
**Hepatic collagen content increased significantly in BDL groups compared to the control and sham groups (P < 0.001)**. There was a difference between liver collagen content of the third group compared to groups 1, 2 and 4 (P < 0.01).

The hepatic collagen content of BDL groups was significantly higher than healthy controls and sham group. Collagen content of the liver with bile duct-ligated was increased, particularly, between control group and group 1 at the 1st week (P < 0.001). The collagen content of the liver was higher at the 2nd week; with a statistically significant difference between control group and group 2. There was no difference between groups 1 and 2 (P > 0.05). At the 3rd week, the collagen content reached to maximum concentration in the Group 3 and compared to other groups (control, group 1, 2, and 4 and sham) (P < 0.001). Moreover, we found a statistically significant decreasing in group 4 at the 4th week (P < 0.01) (Figure 3).

### Histomorphometric collagen measurement

**Figure 4:**
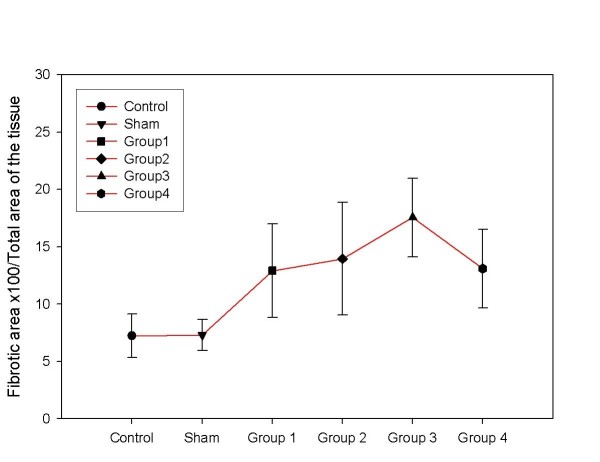
**There was a significant increase in hepatic collagen amount in BDL groups histomorphometricaly compared to the control and sham groups (P < 0.01)**. Collagen peak was found at the end of the third week when compared to the groups 1, 2 and 4 (P < 0.05).

Histomorhometric measurement for collagen demonstrated a very similar pattern as the biochemical collagen measurement. We found that the peak level of collagen was at the third week and a decrease at the fourth week (P < 0.05) (Figure 4).

### Serum levels of AST and ALT

**Figure 5:**
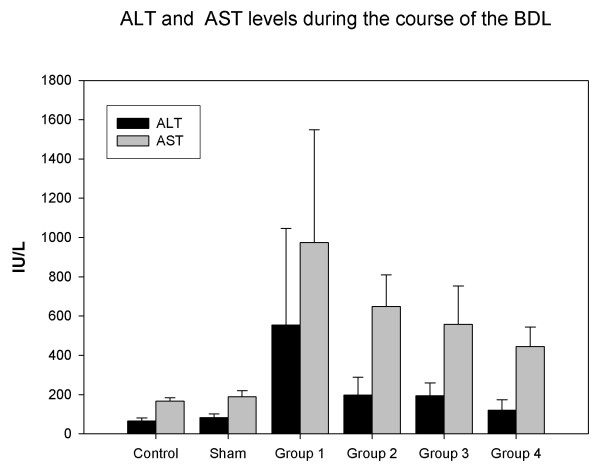
**Serum ALT levels increased in groups 2, 3 and 4 compared to the control and the sham groups: P < 0.01 for groups 2 and 3, P < 0.01 for group 4**. Serum AST levels increased in groups 1, 2, 3 and 4 compared to the control and sham group (P < 0.001).

Serum AST levels increased up to 5 fold of normal at the 1st week. It was started to decrease at the 2nd week, but it was still higher by two fold than control groups at the end of the fourth week (P < 0.001). Serum ALT levels increased up to 10 times at the 1st week (Group 1) when compared with the control group. It started to decrease at the 2nd week. But, serum ALT level was still two times higher than control group at the 4th week (Figure 5).

### Serum bilirubin concentrations

**Figure 6:**
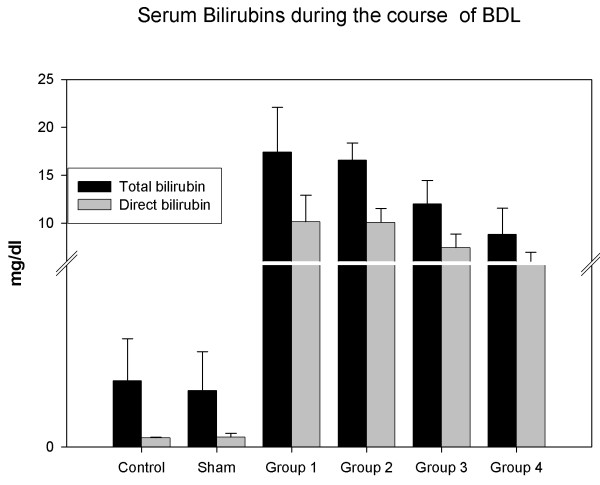
**Serum bilirubin levels increased in groups 1, 2, 3 and 4 compared to the control and sham groups (P < 0.001)**.

Serum total bilirubin level reached to the level of 17.42 mg/dl ± 4.66 (mean ± SD) in group 1 and was almost the same level in group 2 (p > 0.05). The bilirubin level started to decrease in group 3, but it was still high at the 4th week (group 4) (8.81 ± 2.76 mg/dl). Direct bilirubin levels increased in the same manner and 10.15 ± 2.77 mg/dl in Group1. However, there was no statistically significant difference between groups 1 and 2 (P > 0.05). Then, it started to decrease but was still higher at the 4th week (5.28 ± 1.69) (Figure 6).

### Serum levels of ALP and GGT

**Figure 7:**
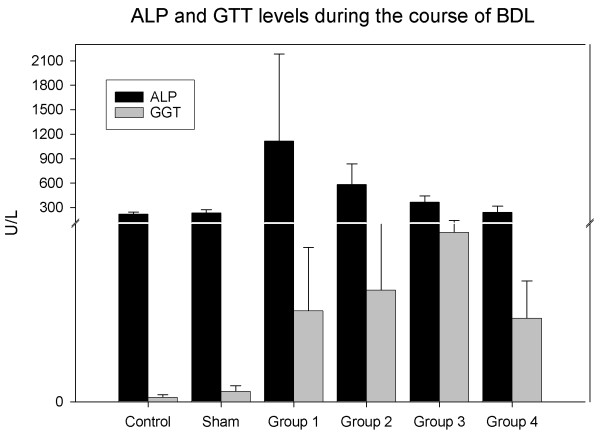
**ALP leves were increased at the end of the first week**. Then, it was decreased to the normal level at the end of the fourth week. The peak level of serum GGT was found at the end of the third week.

Serum ALP level increased up to 5 fold of normal at the 1st week. It started to decrease at the 2nd week. It was still higher at the 4 th week, and no significant difference between control group and group 4 (P > 0.05). Serum GGT level increased up to 20 times and reached to 51.12 ± 35.60. It continued to increase and reached to its maximum concentration at the third week (95.22 ± 48.97). At the end of fourth week it decreased to 47 ± 20. 97 (Figure 7).

## Discussion

There is no animal fibrosis model that completely mimics the spectrum of the diseases seen in human so far [10]. Previously, many investigators used a 4-week bile duct ligated rat model in fibrosis studies [1-18]. Each model facilitated our understanding underlying cellular and molecular mechanisms of fibrogenesis and further assessment of the efficacy of various antifibrotic agents. Our aim was to investigate the quantitative hepatic collagen content with the levels of the serum liver enzymes during the different stages of hepatic fibrosis in bile duct-ligated rats.

In the presented study, we found collagen peak at the third week after bile duct ligation by utilizing both biochemical and histomorphometric methods of hepatic collagen measurement. Since these results were unexpected, we re-validated our results in both groups 3 and 4 by repating examinations. We re-measured the hepatic collagen concentration in revalidation study and found that the hepatic collagen of the third group was higher than the fourth group again. The correlation between liver collagen content of earlier group 3-4 and new group 3-4 was r: 0.92 (P < 0.001).

Serum ALP levels were increased in the first week, then they started to decrease. Finally, serum ALP level was the same as the control group at the fourth week. Similarly, serum GGT level reached its peaks value in the third week and at the end of the fourth week they were still higher. These results were reflecting the changes on the bile ducts in bile ductligated rat model and in a correlation with measured hepatic collegen concentrations. Having these results, we decided to survey the earlier studies to better elucidate who will use this experimental method. Bile duct ligation model has attracted many investigators and many studies in rats as well as other animals performed to clarify this model [1]. The most outstanding and comprehensive study in BDL was performed by Cameron and Oakley in 1932 [2]. They used 120 rats and investigated them from one hour to 9 weeks. They found increased hepatic fibroblastic proliferation starting by day 4 and perilobuler cirrhosis 47 days after bile duct ligation and further frank cirrhosis at the 61st day.

Cameron and Hassan evaluated the structural and functional disturbances in bile ductligated rat and observed them from 1/2 hour to 11 weeks. They found the same structural disturbances as their former study [4]. The serum bilirubin levels were increased to 10.5 mg at the first week which was less than what was observed by us. Then, it was decreased to 4. 8 mg at the 4th weeks, the serum bilirubin fluctuated from week 5 through week 11 in this previous study. Trams et al. evaluated the morphological and functional changes in the liver of rats both after ligation (without division) and excision of the common bile duct [3]. The rats were sacrificed after 1., 3., 14. and 28. days. Fibrous tissue was established around the duct at the 14th and 28th days in CBD excised rats. However, in bile duct-ligated (without division) rats they determined approximately 95% recanalization and normalization of the bile duct. Johnstone et al. reported a quantitave assessment of the structural changes in the rat's liver following bile duct ligation and investigated them from first day to 40 days [19]. Volume proportion of parenchyma, bile duct and supportive stroma were 97/2/1% in sham operated rats, respectively and 37/42/20% respectively at the 40 days after BDL without cirrhosis.

The most controversial area in BDL model is the degree or amount of hepatic fibrosis. Kountauaras et al sacrificed rats 5, 10, 15, and 28 days after BDL and found that all rats had cirrhosis at the 28th day. Several reported frank cirrhosis developed after 3 or 5 weeks [2-12]. On the other hand, there are several studies that did not show any evidence of cirrhosis even at the 40th day following bile duct ligation in the rat [5,20-22]. We did not see cirrhosis in the presented study and our former studies after 28 days bile duct-ligation [23-28]. We observed grades 2 nd 3 fibrosis in our studies. Previously reported that cirrhosis in BDL rats occurs earliest 4-6 weeks after ligation [27,28]. The reason for the differences is unclear and needs further clarification.

## Conclusions

We have shown that fibrosis in bile duct-ligated rats is transient, i.e. reverses spontaneously after 3 weeks both biochemically and histomorphometrically. This contrasts any situation in patients where hepatic fibrosis is progressive and irreversible as countless studies performed by many investigators in the same animal model. In fact, it has been assumed to date, that hepatic fibrosis in patients and animal models progresses to cirrhosis, but does not reverse spontaneously as described in the manuscript.

Although markers of fibrosis and liver damage declined after the 3-week peak, they still remained elevated 4 weeks after ligation, compared to control groups in this study. Thus, this animal model may in fact be suitable to study fibrosis and experimental therapies, provided that correct untreated control groups are included.

Our results are fundamental because many investigators use four-week bile ductligated rats for hepatic fibrosis and antifibrotic therapy studies. To see the effect of antifibrotic therapy on maximum level of collagen is important. Understanding the dynamics of hepatic fibrogenesis we have to use three and four week bile duct-ligated rat models together. We need further studies to clarify the results of the study.

## Competing interests

The authors declare that they have no competing interests.

## Authors' contributions

OT and MB wrote and commented to the paper. OT with NS and GS designed the study. OT, VT, GT and IS performed bile duct ligation and cared animals. CC and GH performed histological grading-staging and histomorphometric measurement. MB performed collagen measurement biochemically. NT and NY commented to the paper. All authors read and approved the final manuscript.

## Pre-publication history

The pre-publication history for this paper can be accessed here:

http://www.biomedcentral.com/1471-230X/11/45/prepub
